# Systemic pro- and anti-inflammatory profiles in acute non-specific low back pain: An exploratory longitudinal study of the relationship to six-month outcome

**DOI:** 10.1371/journal.pone.0287192

**Published:** 2023-06-29

**Authors:** Wei-Ju Chang, Luke C. Jenkins, Peter Humburg, Valerie Wasinger, David M. Walton, Siobhan M. Schabrun

**Affiliations:** 1 Centre for Pain IMPACT, Neuroscience Research Australia (NeuRA), Randwick, New South Wales, Australia; 2 School of Health Sciences, College of Health, Medicine and Wellbeing, University of Newcastle, Callaghan, New South Wales, Australia; 3 School of Health Sciences, Western Sydney University, Penrith, New South Wales, Australia; 4 Stats Central, Mark Wainwright Analytical Centre, UNSW Sydney, Kensington, New South Wales, Australia; 5 Bioanalytical Mass Spectrometry Facility, Mark Wainwright Analytical Centre, UNSW Sydney, Kensington, New South Wales, Australia; 6 School of Physical Therapy, University of Western Ontario, London, Ontario, Canada; 7 The Gray Centre for Mobility and Activity, University of Western Ontario, London, Ontario, Canada; Public Library of Science, UNITED KINGDOM

## Abstract

**Objectives:**

Pro-inflammatory molecules are thought to underpin the development of chronic low back pain (LBP). Although research has begun to explore the association between pro-inflammatory molecules in acute LBP and long-term outcome, no study has explored the role of anti-inflammatory molecules. We aimed to explore whether levels of systemic pro- and anti-inflammatory molecules 1) changed over a period of six months from the onset of acute LBP; 2) differed between people who were recovered (N = 11) and unrecovered (N = 24) from their episode of LBP at six months; 3) baseline psychological factors were related to inflammatory molecule serum concentrations at baseline, three and six months.

**Methods:**

We retrospectively included participants with acute LBP included from a larger prospective trial and examined blood samples for the measurement of pro- and anti-inflammatory molecules and measures of pain, disability, and psychological factors at baseline, three and six months.

**Results:**

The serum concentrations of pro- and anti-inflammatory molecules did not differ over time when compared between participants who recovered and those who did not recover at six-month follow-up. At three months, the unrecovered group had higher interleukin (IL)-8 and IL-10 serum concentrations than the recovered group. Baseline psychological factors were not related to inflammatory molecules at any time point.

**Discussion:**

This exploratory study showed that levels of systemic inflammatory molecules did not change over the course of LBP, irrespective of whether people were recovered or unrecovered at six months. There was no relationship between acute-stage psychological factors and systemic inflammatory molecules. Further investigation is needed to elucidate the contribution of pro- and anti-inflammatory molecules to long-term LBP outcome.

## Introduction

Approximately 85% of low back pain (LBP) has no identifiable cause (termed non-specific LBP) [1]. Chronic non-specific LBP is characterised by complexity and heterogeneity in biological and psychosocial domains [2]. Although psychological factors are currently the best prognostic indicators for LBP, they explain only a small proportion (~24%) of variance in outcomes [3–5], highlighting the likely contribution of other factors to the development of chronic LBP. Systemic inflammatory molecules have been previously associated with LBP severity [6–8], and have emerged as one factor that could contribute to long-term LBP outcome [9].

Systemic pro-inflammatory molecules have been investigated in LBP of various durations and clinical diagnoses [10–14]. For example, research has shown associations between discrete pro-inflammatory cytokine profiles in acute LBP and long-term outcome. Specifically, acute-stage tumour necrosis factor-α (TNF-α) was elevated in people reporting ongoing LBP at six months while C-reactive protein (CRP) and interleukin-6 (IL-6) were elevated in those who recovered [9]. In chronic LBP, serum concentrations of pro-inflammatory molecules including CRP, interleukin-1ß (IL-1ß), IL-6 and TNF-α were elevated in people with intervertebral disc pathologies compared with pain-free controls [9, 15–21]. Similar findings have also been reported in chronic non-specific LBP when the pathoanatomical source of pain could not be identified [10, 22]. Although it is unclear why pro-inflammatory molecules are elevated in chronic LBP, sterile inflammation of injured tissues triggered via action of pro-inflammatory molecules is one plausible hypothesis [23]. Alternatively, interaction between systemic inflammatory molecules and psychological comorbidities could influence LBP outcome. Indeed, depressive symptoms combined with elevated TNF-α in acute LBP have been shown to be associated with poor recovery at six months [9]. Thus, systemic inflammatory molecules could contribute to the development of chronic LBP although the nature of the relationship is unknown.

Anti-inflammatory molecules such as interleukin-4 (IL-4), interleukin-10 (IL-10) and transforming growth factor-β1 (TGF-β1) are known to downregulate pro-inflammatory molecules [24]. However, their involvement in the development of chronic LBP is unclear and research is scarce. Evidence has shown higher IL-4 and IL-10 serum concentrations in people with mild lumbar radicular pain than those with severe pain and pain-free controls [25]. Animal studies report TGF-β1 can supress inflammation in disc pathologies [26, 27]. Results from people with mixed musculoskeletal trauma, including some with LBP, revealed a cross-sectional association between acute pain severity and IL-10 and TGF-β1, moderated by social variables or psychiatric comorbidities [28]. Further, cross-sectional studies suggest an imbalance between pro- and anti-inflammatory molecules (lower anti-inflammatory and higher pro-inflammatory molecules) in chronic LBP, leading authors to hypothesise that this imbalance reflects a pathophysiological mechanism underlying the development of chronic LBP [13, 25, 29]. However, whether systemic anti-inflammatory molecules change over the course of LBP or contribute to the development of chronic symptoms is unknown.

Using data from the UPWaRD (Understanding Persistent pain Where it ResiDes) prospective longitudinal cohort study, we aimed to explore whether levels of systemic pro- and anti-inflammatory molecules 1) changed over a period of six months from the onset of acute LBP; 2) differed between people who were recovered and unrecovered from their episode of LBP at six months; 3) baseline psychological factors were related to systemic pro- and anti-inflammatory molecules at baseline, three and six months.

## Materials and methods

### Study design, setting and participants

Thirty-five participants with acute LBP whose blood samples were collected at three timepoints (baseline, three and six months) from the UPWaRD study were included in this longitudinal study. The UPWaRD study aimed to investigate whether sensorimotor cortex activity, genetic and psychosocial factors in the acute stage of LBP predict outcome at six months (Trial Registration Number ACTRN12619000002189) and was conducted between December 2014 and July 2019 at Neuroscience Research Australia and Western Sydney University in Sydney, Australia [30, 31]. Participants were recruited from the community through newspaper/on-line advertisements, flyers and social media sites, primary health care professionals (i.e. general practitioners and physiotherapists), and local hospitals in South East Sydney and South Western Sydney local health districts, New South Wales, Australia. Ethical approval was obtained from Neuroscience Research Australia (SSA: 16/002) and the Western Sydney University Human Research Ethics Committee (H10465). All participants received reimbursement for their time and travel expenses consistent with our standard ethical protocols.

### Eligibility criteria

Eligible participants were 18 years of age or older and currently experiencing acute non-specific LBP [32]. Acute LBP was defined as pain located between the lower border of the 12^th^ ribs and the gluteal fold, that lasted for more than 24 hours and less than six weeks, preceded by a period of at least one month without LBP [32–34]. Participants remained eligible if they reported leg pain that was not radicular pain resulted from neural tissue involvement or lumbosacral radiculopathy. Radicular pain was suspected if participants reported radiating leg pain, leg pain worse than back pain, worsening leg pain during coughing, sneezing or straining [35], and a positive straight leg raise test [36]. Lumbosacral radiculopathy was suspected if the participant had muscle weakness, loss of sensation, or loss of reflexes corresponding to a particular nerve root, or a combination of these [36]. Participants with suspected radicular pain and/or lumbosacral radiculopathy were excluded during the clinical examination in the study. All participants were required to speak and read English adequately to understand the information on the consent form of the study and instructions from the researchers. Any people who presented with suspected serious spine pathology (i.e. fracture, tumour, cauda equina syndrome), other major diseases/disorders (i.e. schizophrenia, chronic renal disorder, multiple sclerosis), a history of spine surgery, any other chronic pain conditions or contraindications to the use of transcranial magnetic stimulation were excluded [37]. Further, although participants in the UPWaRD study were allowed to take medications for their acute LBP (i.e. analgesics, non-steroid anti-inflammatory drugs [NSAIDs]), those who took NSAIDs were excluded from this exploratory study. Participants who did not have blood samples at all three timepoints were also excluded.

### Procedures

Potential participants were contacted via telephone to determine their eligibility and invite participation. Written informed consent was obtained from each participant upon their arrival at the baseline assessment. Participants were assessed within six weeks of acute LBP onset (baseline) and at three- and six-month follow-up. Data for demographics were collected at baseline. The use of health care: 1) number of visits to general practitioner; 2) number of visits to allied health care professionals (i.e. physiotherapy or chiropractor) and 3) pain medication: use of opioids or non-opioid analgesics such as paracetamol were recorded for each participant at baseline.

### Measures of inflammatory molecules

Peripheral venous blood (~ 8ml) was drawn into serum tubes (BD Vacutainer, SST II Advance), inverted four to five times, and clotted at room temperature for 30 min. Serum was then separated by centrifugation (2500 rpm, 15 min) and stored in approximately 450μL aliquots at -80°C until measurement. Serum concentrations of CRP, TNF-α, interleukins (IL-1ß, IL-2, IL-4, IL-6, IL-8, IL-10, IL-15) and TGF-β1 were measured using an enzyme-linked immunosorbent assay (ELISA) (Protein Simple-Simple Plex Cartridge Kit, BioTechne, California USA). Samples were prepared and loaded into the cartridge according to a standard procedure provided by the manufacturers (BioTechne, CA, USA) with all steps in the immunoassay procedure automated by the SimplePlex^TM^ platform (BioTechne). Cartridges included a logistic weighted standard curve with an average of five replicates of each value. Single data (pg/mL) for each sample were automatically calculated in triplicate from the three glass nanoreactors per sample. All ranges for detection and quantification are provided in detail for each of the proteins evaluated from the company website documentation. Briefly, the detection limits for each blood biomarker were as follows: CRP: 1.24 pg/ml, TNF-α: 0.278 pg/ml, IL-1β: 0.064 pg/ml, IL-2: 0.18 pg/ml, IL-4: 0.05 pg/ml, IL-6: 0.26 pg/ml, IL-8: 0.08 pg/ml, IL-10: 0.14 pg/ml, IL-15: 0.193 pg/ml and TGF-β1: 5.29 pg/ml. Similarly, the upper limits and range of quantification were approximately 4 orders of magnitude concentration. Inter-assay coefficients of variation were less than 10% [38]. Values below the test sensitivity were set to zero. All protein specific cartridges were also confirmed for accuracy using a known concentration of protein as an additional measure of reliability. All procedures were conducted by trained researchers experienced with these methods.

### Measures of pain and disability

Participants were asked to score their pain on average over the previous week and their pain at the time of testing using an 11-point numerical rating scale (NRS [0 = ‘no pain’, 10 = ‘worst pain imaginable’]). *The Roland Morris Disability Questionnaire (RMDQ)*, including 24 questions, was used to assess the level of disability experienced as the result of LBP [39]. A score of ‘0’ indicated no LBP-related disability and a score of ‘24’ indicated severe disability.

### Measures of psychological factors

Psychological factors were assessed using: *1) The Pain Catastrophising Scale*- a 13-item self-report instrument to assess patients’ thoughts and feelings about pain in the domains of magnification, rumination and helplessness (three subscales) that has shown adequate evidence of reliability and validity for use in LBP populations [40]. *2) The Depression Anxiety and Stress Scale- 21 (DASS 21)*—a 21-item self-administered questionnaire to measure negative emotional states of depression, anxiety and stress [41]. Higher scores in the subscales indicate more severe condition of depression, anxiety and stress [42]. 3) *The Pain Self Efficacy Questionnaire (PSEQ)*- a 10-item, 7-point (0 to 6) instrument that assesses an individual’s confidence performing activities while in pain [43]. Scores range from 0 to 60, with a clinical cut-off value < 40 (where scores below 40 represent low pain self-efficacy) [44]. The PSEQ has sound evidence of high internal reliability and validity [45].

### Outcome measures

The primary outcome measure was the presence of LBP at six-month follow-up defined as i) LBP on average over the previous week ≥ 1 (out of 10) on a NRS [46, 47] or ii) an LBP-related disability score of ≥ 3 (out of 24) on the RMDQ [48]. Participants reporting LBP at six months were considered “unrecovered” from their acute episode of LBP. These cut-off scores on the NRS and the RMDQ have been used in previous studies as they are considered to accurately classify participants as unrecovered from LBP [48–50].

### Sample size

It has been suggested that a statistical approach to determine the sample size is not appropriate in studies using non-probability samples and the “rule of thumb’ recommends 15 to 30 participants per group in experimental studies using non-probability samples [51]. Our sample size is consistent with this recommendation.

### Statistical analysis

All statistical analyses were conducted using R, version 4.0.3 (R Development Core Team, Vienna, Austria) [52]. Demographics and baseline characteristics of participants are presented as mean and standard deviation (SD) or median and interquartile range (IQR) as appropriate for continuous variables and as frequency (percentage) for categorical variables. IL-2 and IL-4 serum concentration were excluded from the analyses as no participants had values higher than test sensitivity (value = zero) in this retrospectively selected sample. The use of analgesics was converted to a binary variable (“yes” or “no”).

Linear mixed-effects models were used to examine changes in serum concentrations of pro- and anti-inflammatory molecules over three time points (*time*: baseline vs. three months vs. six months) between LBP recovery status at six months (*group*: recovered vs. unrecovered) (R package *lme4*) [53]. A separate model was fitted for each inflammatory molecule. Independent variables included group, time, and the group x time interaction term as fixed effects and participant-specific random intercepts. Residual plots were visually inspected to confirm no obvious deviations from homoscedasticity or normality. To determine whether the group x time interactions were significant, analysis of variance (ANOVA) was used to compare the models with and without the group x time interaction term. Between-model differences (p < 0.05) indicate significant group x time interactions for inflammatory molecules. Similarly, the significance of group or time main effects was assessed using ANOVA to compare the models including the group x time interaction term vs the models excluding group or time accordingly. A sensitivity analysis was conducted to determine whether participants with severe pain (NRS≥7) at baseline influenced the results. The analyses were repeated after excluding three participants with severe acute LBP. Additionally, pairwise comparisons between groups and time points were also conducted. Sidak adjustment for multiple testing was used as appropriate to correct the p values accordingly. Changes in pain, disability and psychological factors over time between recovered and unrecovered participants were also compared using this approach.

To explore whether baseline psychological factors were related to serum concentrations of pro- and anti-inflammatory molecules at baseline, three and six months, principal component analysis (PCA) and multiple regression were used. Baseline psychological factors (scores of DASS 21- depression, anxiety, stress subscales, PSEQ, PCS- rumination, magnification, helplessness subscales) were entered into a PCA to identify clusters of linear, noncorrelated principal components (PCs) (R package *factoextra*) [54]. The first PC (PC1) explains the largest proportion of the variability in the data, followed by the second PC (PC2) and so on [55]. Variables with a factor loading > 0.4 or < -0.4 were determined as having considerable influence on each PC. To construct a linear regression model, PCs with eigenvalues > 1 were selected as candidate predictor variables and the serum concentration of each inflammatory molecule at each time point as the dependent variable [56]. Sidak adjustment for multiple testing was used to correct the p values of the linear regression models.

## Results

### Participant baseline characteristics

Twenty-four participants (68.6%) had LBP and 11 (31.4%) had no LBP at six-month follow-up. Baseline participant characteristics are summarised in Table 1. Those who reported LBP at six months were older (W = 0.93, p = 0.007) and reported more visits to their general practitioner (W = 88.00, p = 0.035) at baseline than those who recovered. There were no between-group differences in any other baseline characteristics (all p>0.12).

**Table 1:** Baseline participant characteristics for the recovered and unrecovered groups.

	Recovered (N = 11)	Unrecovered (N = 24)	*P* value
**Age (year)**	32.7 (11.6)	47.8 (17.8)	**<0.01**
**Sex (N)**	Female = 5 (45.5%)	Female = 13 (54.2%)	0.65
	Male = 6 (54.5%)	Male = 11 (45.8%)	
**Body mass index (kg/m** ^**2**^ **)**	25.2 (4.1)	23.5 (7.2)	0.28
**Highest education- Tertiary degree/above (N)**	8 (72.7%)	10 (41.7%)	0.12
**Average pain in the last week (0–10 on NRS)**	2.8 (1.5)	4.3 (1.7)	0.19
**RMDQ**	3.7 (2.6)	5.7 (4.6)	0.43
**First episode of LBP (N)**	3 (27.3%)	4 (16.7%)	0.53
**Constant LBP with fluctuating pain (N)**	4 (36.4%)	13 (54.2%)	0.22
**Worst side of LBP (N)**	Left = 7 (63.6%)	Left = 11 (45.8%)	0.35
	Right = 4 (36.4%)	Right = 13 (54.2%)	
**Number of visiting to general practitioner (N)**	0 (0.0)	0.6 (1.0)	**0.04**
**Number of visiting to allied health care (N)**	1.6 (3.0)	2.1 (3.7)	0.88
**Use of analgesics (N)**	0 (0.0%)	2 (8.3%)	0.35

Continuous data are described as mean and standard deviation. Categorical data are described as number (%). Variable means were compared between recovered and unrecovered low back pain participants using analysis of variance (continuous variables) or Kruskal-Wallis rank sum tests (categorical variables). Significant values are in bold font. Note: LBP- low back pain; NRS- numeric rating scale (0 = no pain and 10 = the worst pain imaginable); NSAIDs- non-steroid anti-inflammatory drugs; RMDQ- Roland-Morrison Disability Questionnaire.

### Systemic inflammatory molecules did not change over time, regardless of LBP recovery status

**Fig 1:**
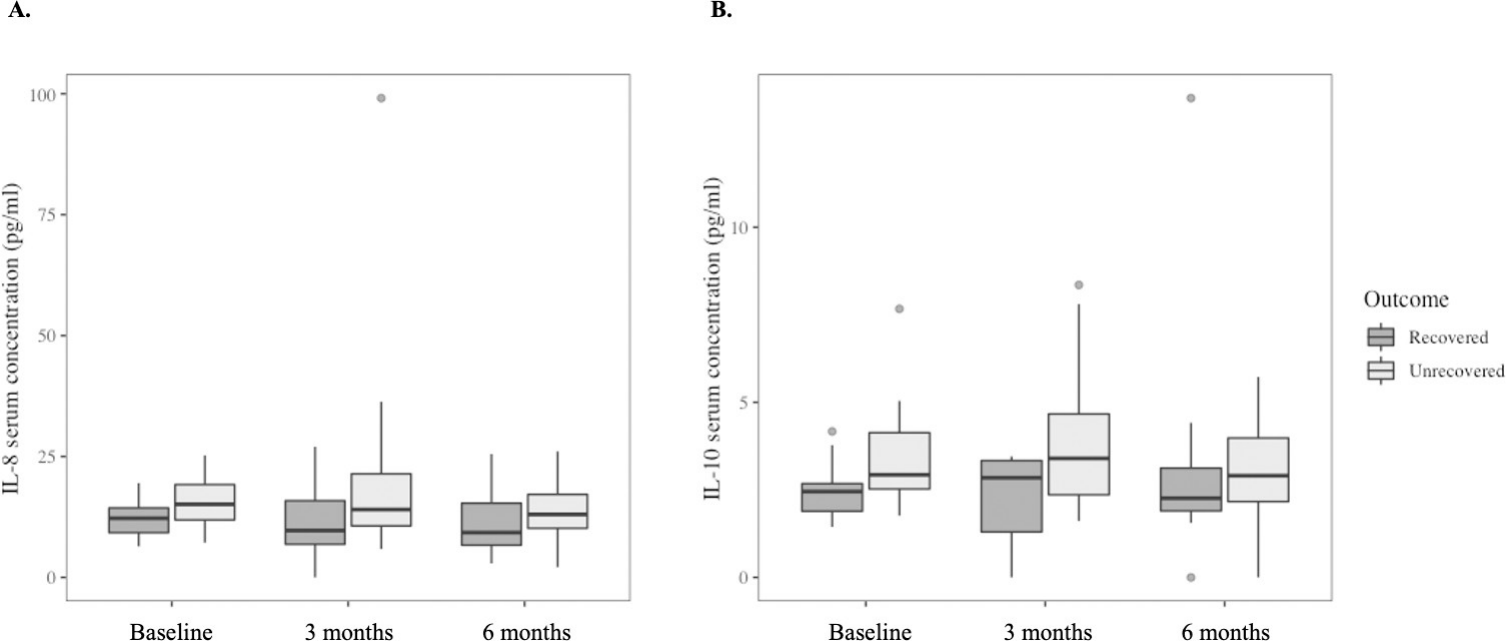
Serum concentrations of IL-8 (panel A) and IL-10 (panel B) at baseline, three- and six-month follow-ups in participants who did and did not recover at six months. Box plots represent median (horizontal line), 25^th^ and 75^th^ percentiles (box), and the ranges for the bottom and top 5^th^ percentiles of the data values (outside the box). Linear mixed-effects models showed IL-8 and IL-10 levels (p<0.05) were higher in the unrecovered group than the recovered group at three months. IL = interleukin.

There were no group x time interactions for serum concentrations of inflammatory molecules (Table 2). Pairwise comparisons showed that serum concentrations of IL-8 (adjusted p = 0.043) and IL-10 (adjusted p = 0.030) were higher in the unrecovered group than the recovered group at three months (Fig 1). Data are provided in S1 Table. After excluding three participants with severe pain (NRS≥7) at baseline, the tests were repeated. The findings showed the same between-group differences in IL-8 and IL-10 serum concentrations at three months, indicating that those with severe pain did not influence the results.

**Table 2:** Linear mixed-effects model results of serum concentrations of inflammatory molecules, pain and disability, and psychological factors between the recovered and unrecovered LBP groups over time (baseline, three and six months).

	Group Effect		Time Effect		Group x Time Effect	
	Chi^2^	P value	Chi^2^	P value	Chi^2^	P value
***Systemic inflammatory molecules***						
**IL-1β**	0.80	0.85	2.04	0.73	0.72	0.70
**IL-6**	1.71	0.63	0.69	0.95	0.20	0.90
**IL-8**	4.88	0.18	4.27	0.37	1.39	0.50
**IL-10**	8.10	**0.04**	5.78	0.22	5.48	0.06
**IL-15**	3.38	0.34	3.1	0.46	3.14	0.21
**TNF-α**	2.23	0.53	3.72	0.45	0.58	0.75
**CRP**	1.87	0.60	6.48	0.17	1.85	0.40
**TGF-β1**	1.72	0.63	2/61	0.62	1.55	0.46
***Pain and disability***						
**NRS**	21.96	**<0.01**	47.54	**<0.01**	4.84	0.09
**RMDQ**	9.98	**0.02**	19.82	**<0.01**	1.98	0.37
***Psychological factors***						
**DASS 21 -Depression**	4.05	0.26	3.19	0.53	0.23	0.89
**DASS 21 -Anxiety**	8.54	**0.04**	1.28	0.87	0.28	0.88
**DASS 21 -Stress**	5.93	0.12	2.00	0.74	1.93	0.38
**PSEQ**	11.83	**0.01**	6.62	0.16	2.33	0.31
**PCS Rumination**	4.40	0.22	8.74	0.07	2.74	0.25
**PCS Magnification**	3.58	0.31	8.61	0.07	0.84	0.66
**PCS Helplessness**	4.53	0.21	10.44	**0.03**	2.72	0.26

Measures of systemic inflammatory molecules, pain, disability, and psychological factors were compared between groups (recovered vs unrecovered) over time (baseline, three and six months). The significance of the group x time interactions was determined by comparing the models with and without the group x time interaction term using Chi^2^ analyses. Using the same approach, the significance of group or time main effects was assessed by comparing the models including the group x time interaction term vs the models excluding group or time accordingly. Bold fonts indicate significant values (P<0.05). Note: CRP- C-reactive protein; DASS 21- The Depression Anxiety Stress Scale- 21; IL- interleukin; NRS- numeric rating scale; PCS- Pain Catastrophising Scale; PSEQ- Pain Self-Efficacy Questionnaire; Roland Morris Disability Questionnaire- RMDQ; TNF-α- tumour necrosis factor- α; TGF-β1- transforming growth factor-β1.

### Pain and disability improved in both the recovered and the unrecovered groups over time

**Fig 2:**
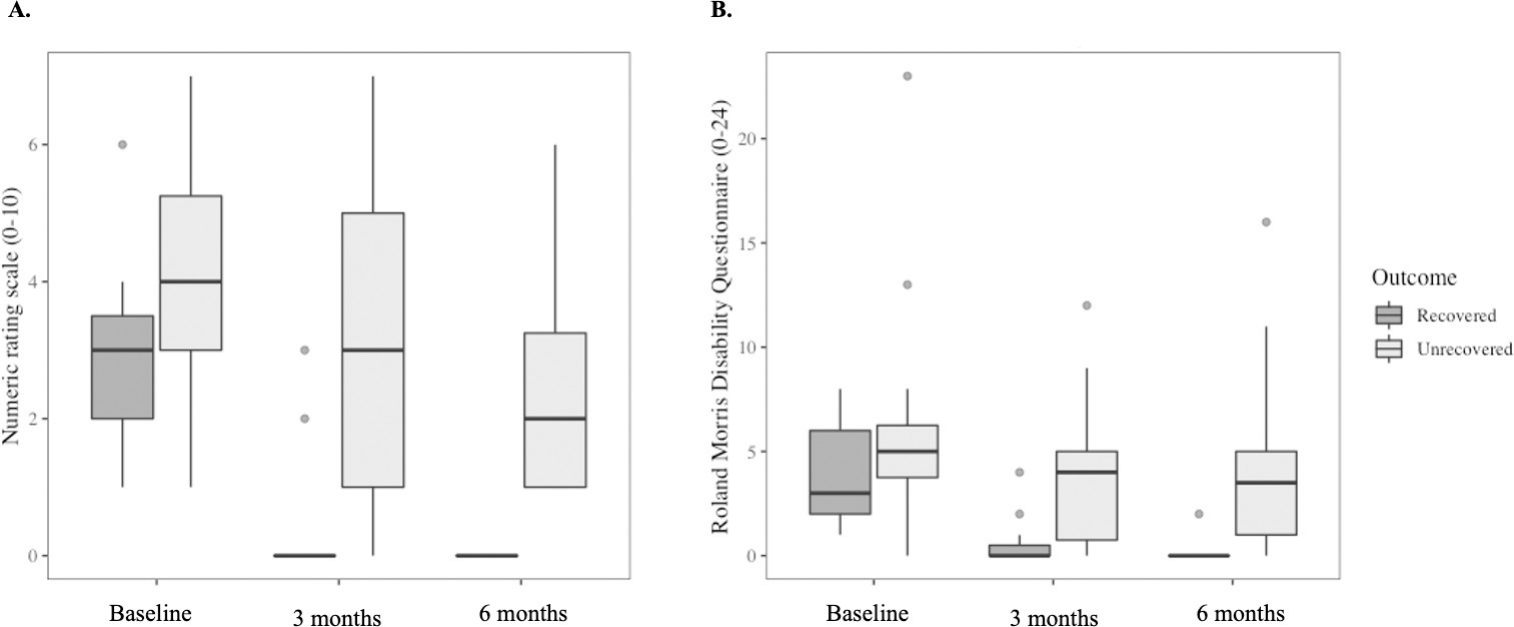
Pain (panel A; measured by a 0–10 numeric rating scale) and disability (panel B; measured by Roland Morrison Disability Questionnaire) at baseline, three and six months in participants who did and did not recover at six months. Box plots represent median (horizontal line), 25^th^ and 75^th^ percentiles (box), and the ranges for the bottom and top 5^th^ percentiles of the data values (outside the box). Linear mixed-effects models showed pain and disability reduced at three months compared with baseline and remained stable at six months in both groups. Pain and disability were significantly lower in the recovered group than the unrecovered group at three and six months.

Despite no group x time interactions for pain or disability, there were main effects of group and time for pain and disability (Table 2). At baseline, pain was higher in the unrecovered group than the recovered group (adjusted p<0.017), whereas disability did not differ (adjusted p = 0.126). Pain (adjusted p<0.003) and disability (adjusted p<0.031) decreased from baseline to three months and were unchanged between three and six months (adjusted p>0.293) in both groups. Pain and disability were higher in the unrecovered group than the recovered group at three (adjusted p<0.015) and six months (adjusted p<0.004) (Fig 2).

### Psychological profiles differ between the recovered and the unrecovered groups

**Fig 3:**
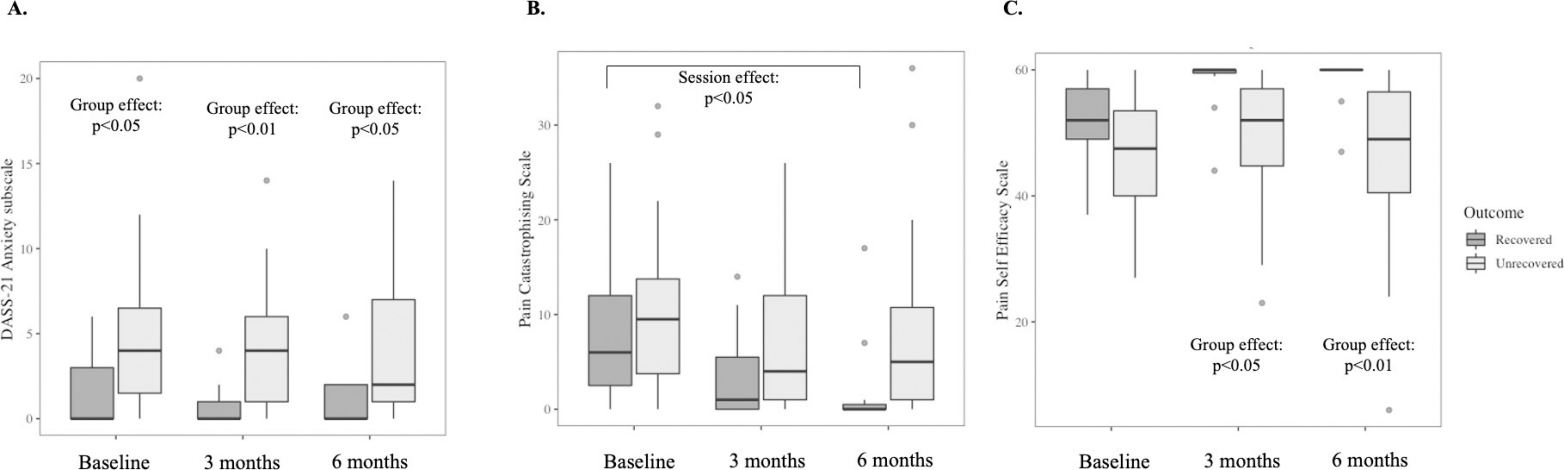
Anxiety (panel A; measured by DASS-21 Anxiety subscale), pain catastrophising (panel B; measured by Pain Catastrophising Scale total score) and pain self-efficacy (panel C; measured by Pain Self- Efficacy Scale) at baseline, three and six months in participants who did and did not recover at six months. Box plots represent median (horizontal line), 25^th^ and 75^th^ percentiles (box), and the ranges for the bottom and top 5^th^ percentiles of the data values (outside the box). Significant group and session effects derived from linear mixed-effects model are depicted.

Although there were no group x time interactions for psychological factors, there was a main effect of group for anxiety and pain self-efficacy, and a main effect of time for pain catastrophising- helplessness (Table 2). The unrecovered group had higher levels of anxiety at all time points (adjusted p<0.029) (Fig 3A) and a higher level of stress at three months (adjusted p = 0.021) than the recovered group. Pain catastrophising- rumination and helplessness (adjusted p<0.048) reduced from baseline to six months only in the recovered group (Fig 3B). Pain self-efficacy was similar in both groups at baseline (adjusted p = 0.114) but lower in the unrecovered than the recovered group at three (adjusted p = 0.021) and six months (adjusted p = 0.002) (Fig 3C).

### Baseline psychological factors were not related to systemic inflammatory molecules

The first principal component (PC1) was the only principal component selected as a predictor variable in the regression analyses (S2 Table). Loading of the psychological factors on PC1 is summarised in Table 3. There was no association between PC1 and systemic inflammatory molecules at any time point, with the exception of IL-6 at baseline (p = 0.004) and CRP at three months (P = 0.027) (Table 4). However, after adjustment for multiple comparisons, these associations were no longer significant (adjusted p>0.096).

**Table 3:** Loading factor of the psychological factors in the principal components with eigenvalue > 1 at baseline.

	Baseline
	PC1
DASS 21 –Depression	-0.77
DASS 21 –Anxiety	-0.79
DASS 21 –Stress	-0.90
PSEQ	0.67
PCS–Rumination	-0.82
PCS–Magnification	-0.85
PCS–Helplessness	-0.91

Psychological factors with a loading factor > 0.4 or < -0.4 (in bold) have considerable influence on each principal component. Note: DASS 21- The Depression Anxiety Stress Scale- 21; PCS- Pain Catastrophising Scale; PSEQ- Pain Self-Efficacy Questionnaire.

**Table 4:** Coefficients of the linear regression models for the relationships between the first principal component (PC1) at baseline and serum concentrations of the inflammatory biomarkers at baseline, three and six months.

	Baseline	Three months	Six months
	PC1	PC1	PC1
**IL-1β**	0.02	0.24	-0.02
**IL-6**	-0.47*	-0.23	-0.02
**IL-8**	-0.24	0.09	-0.02
**IL-10**	0.11	0.08	0.06
**IL-15**	0.04	0.06	0.21
**TNF-α**	-0.06	0.08	0.22
**CRP**	0.03	-0.37^†^	-0.30
**TGF-β1**	-0.001	-0.30	-0.005

Note

*Unadjusted p value < 0.01

^†^Unadjusted p value < 0.05; CRP- C-reactive protein; IL- interleukin; PCS- Pain Catastrophising Scale; TNF-α- tumour necrosis factor- α; TGF-β1- transforming growth factor-β1.

## Discussion

This exploratory study found that serum concentrations of pro- and anti-inflammatory molecules did not change from the acute stage of LBP to six-month follow-up, irrespective of LBP recovery status. However, at three-month follow-up, serum concentrations of IL-8 and IL-10 were higher in the unrecovered than the recovered participants. The trajectories of acute LBP intensity, LBP-related disability and pain catastrophising differed between the recovered and the unrecovered groups. Baseline psychological factors were not related to serum concentrations of systemic inflammatory molecules at any time point.

Despite reductions in pain and disability in both the recovered and unrecovered groups, serum concentrations of pro- and anti-inflammatory molecules did not change over time, suggesting that systemic inflammatory molecules might not influence the trajectories of acute LBP or LBP-related disability. In contrast, a recent report in early acute LBP (within two weeks of onset) showed a higher serum concentration of CRP in the recovered than the unrecovered group at baseline and a lower serum concentration of TNF-α in the recovered than the unrecovered group at baseline and six months [9]. Differences in the inclusion criteria for acute LBP (two vs. six weeks from onset), follow-up time points (without vs. with three-month follow-up), definition of LBP outcome (unrecovered/partially recovered/recovered vs. unrecovered/recovered) and sample size (N = 109 vs. 35) could explain the conflicting results. The variation in the temporal profile of systemic inflammatory molecules in acute non-specific LBP and its contribution to the recovery of acute LBP require further investigation.

Concurrently higher IL-8 and IL-10 serum concentrations in the unrecovered than the recovered group at three months were unexpected. Traditionally, IL-8 (pro-inflammatory) and IL-10 (anti-inflammatory) are considered as having antagonistic inflammatory effects. However, recent evidence suggests that IL-8 can also act as an anti-inflammatory molecule under specific circumstances [57, 58], and IL-10 can also exert pro-inflammatory effects [59]. The pro-inflammation property of IL-10 has been reported in conditions such as irritable bowel syndrome, cancer and severe SARS-CoV-2 infection [60, 61]. It is unclear why IL-8 and IL-10 were concurrently elevated at three months in those unrecovered from LBP, although their dual role in inflammatory function might explain this finding [58, 62]. As the properties of inflammatory molecules are determined by factors that were not examined in this study (i.e. cytokine levels, nature of target cell and activating signal, timing/sequence of cytokine action) [63], the actual inflammatory function of IL-8 and IL-10 cannot be determined in this cohort. Nevertheless, a key finding from this study is that the simple dichotomisation of inflammatory molecules into two opposite classes cannot explain the complex inflammatory actions occurring in conditions such as LBP.

Compared with the recovered group, the unrecovered group had consistently higher levels of anxiety, lower pain self- efficacy and no improvement in pain catastrophising over time. However, psychological factors in acute LBP were not associated with serum concentrations of systemic inflammatory molecules. Our findings conflict with previous research showing that high levels of depression are associated with elevated systemic IL-6 [64], and greater pain rumination is associated with low systemic TNF levels [65], suggesting a heterogeneous association between systemic pro-inflammatory molecules and psychological factors in acute LBP. Further, the relationship between anti-inflammatory molecules and psychological factors in LBP remains unclear, despite early evidence for an association between anti-inflammatory molecules and psychological distress [66–68]. Although we found no evidence of a relationship between anti-inflammatory molecules and psychological factors in acute LBP, it is conceivable that the relationship between clinical symptoms and systemic inflammatory molecules is more complex than a simple binary association [28]. Indeed, socioeconomical status and pre-existing life stress are reported to moderate the association between systemic inflammation and pain severity in acute musculoskeletal disorders [28]. Due to the relatively small sample size, the effects of social and environmental factors on our data were not explored.

This study has some limitations. First, we retrospectively included participants from a larger cohort only if their blood samples were available at all three time points. Consistent with previous research [65], participants taking NSAIDs at baseline (N = 4) were excluded as these medications could influence levels of inflammation [69]. However, understanding the interaction between LBP, medications such as NSAIDs and inflammatory molecules is an important area that needs further investigation. Moreover, the numbers of participants in the recovered and unrecovered groups were uneven. As these limitations could reduce the power of this study and increase the likelihood of false negative findings, the findings require replication in larger samples. Second, we did not include pain-free controls. Although systemic inflammatory molecules did not differ based on participants’ recovery status, except for IL-8 and IL-10 at three months, whether they differ in different stages of LBP cannot be determined in this study. Third, only three participants were considered as having severe pain (NRS ≥ 7) [70] at baseline, limiting the generalisation of our findings to people with mild to moderate acute LBP. Fourth, while this study applied a commonly used definition of acute LBP (pain lasting for less than six weeks) [32–34], it is plausible that systemic inflammatory profiles differ between people with different durations of acute LBP. Lastly, factors related to blood sample collection (i.e. time of day when blood samples were drawn, stress and physical activity level, food digestion or fasting) that were not controlled in this study could influence our findings [71]. Further, the relationship between the plasma and joint fluid levels of inflammatory molecules could not be assessed in this study because the structural cause of pain could not be identified in our cohort of non-specific LBP [1]. As animal studies have suggested an association between joint inflammation and elevated systemic pro-inflammatory molecules [69], this area requires further investigation in humans.

### Conclusion

This exploratory study showed that systemic inflammatory molecules did not change over the course of LBP, regardless of whether people were recovered or unrecovered at six months. There was no relationship between acute-stage psychological factors and systemic inflammatory molecules. Further investigation is needed to elucidate the contribution of pro- and anti-inflammatory molecules to long-term LBP outcome.

## Supporting information

S1 Table Group data (mean and standard deviation) for pain, disability, psychological distress and serum concentrations of inflammatory blood markers measured at baseline, three and six months. (DOCX)

S2 Table Eigenvalues and the degrees of variance (%) of the principal components based on psychological factors at baseline. (DOCX)
