# Supplementary material for: Systemic pro- and anti-inflammatory profiles in acute non-specific low back pain: An exploratory longitudinal study of the relationship to six-month outcome
Source: PLoS One. 2023 Jun 29;18(6):e0287192. doi: 10.1371/journal.pone.0287192 (PMC10309993; doi:10.1371/journal.pone.0287192)
Supplement: S1 Table — (DOCX) [file pone.0287192.s001.docx]

**Supplementary TABLE S1. Group data (mean and standard deviation) for pain, disability, psychological distress and serum concentrations of inflammatory blood markers measured at baseline, three and six months.**

|  | **Baseline** | | **Three months** | | **Six months** | |
| --- | --- | --- | --- | --- | --- | --- |
|  | **Recovered** | **Unrecovered** | **Recovered** | **Unrecovered** | **Recovered** | **Unrecovered** |
| **Pain** | 3.6 (2.4) | 4.4 (1.6) | 0.4 (1.0) | 3.0 (2.0) | 0.0 (0.0) | 2.6 (1.6) |
| **RMDQ** | 4.9 (4.0) | 5.8 (4.4) | 0.5 (1.2) | 3.8 (3.3) | 0.2 (0.6) | 3.9 (3.8) |
| **DASS 21 Depression** | 1.9 (2.8) | 5.9 (7.9) | 1.4 (3.6) | 4.4 (5.4) | 2.3 (3.9) | 5.2 (5.3) |
| **DASS 21 Anxiety** | 1.4 (2.1) | 4.5 (4.7) | 0.8 (1.3) | 4.2 (3.8) | 1.5 (2.6) | 3.9 (4.1) |
| **DASS 21 Stress** | 5.1 (5.3) | 9.9 (8.2) | 4.3 (4.5) | 9.4 (8.5) | 6.0 (6.2) | 8.3 (6.7) |
| **PSEQ** | 52.3 (6.6) | 45.4 (10.6) | 58.2 (1.8) | 48.5 (3.5) | 58.6 (3.8) | 46.3 (13.3) |
| **PCS Rumination** | 3.0 (3.1) | 3.3 (2.9) | 1.2 (1.8) | 2.7 (3.5) | 0.6 (1.7) | 2.7 (3.3) |
| **PCS Magnification** | 1.5 (2.0) | 2.4 (2.2) | 0.6 (1.2) | 1.7 (1.9) | 0.5 (1.2) | 1.8 (2.3) |
| **PCS Helplessness** | 3.2 (3.9) | 4.3 (4.0) | 1.4 (2.3) | 2.8 (3.4) | 0.8 (2.1) | 3.7 (4.2) |
| **IL-1β (pg/ml)** | 0.5 (1.0) | 0.3 (0.7) | 0.6 (1.5) | 0.4 (0.8) | 0.4 (0.4) | 0.6 (1.0) |
| **IL-6 (pg/ml)** | 1.8 (1.3) | 2.3 (3.3) | 1.4 (1.0) | 2.6 (2.2) | 1.0 (1.2) | 2.2 (3.5) |
| **IL-8 (pg/ml)** | 11.8 (3.7) | 15.6 (4.9) | 12.0 (7.2) | 19.0 (17.6) | 11.4 (6.5) | 14.0 (5.6) |
| **IL-10 (pg/ml)** | 2.5 (0.8) | 3.4 (1.2) | 2.5 (1.5) | 3.8 (1.8) | 3.3 (3.3) | 3.2 (1.3) |
| **IL-15 (pg/ml)** | 3.1 (1.7) | 3.3 (0.9) | 2.8 (1.8) | 3.8 (3.5) | 3.5 (2.4) | 3.0 (0.9) |
| **TNF-α (pg/ml)** | 7.4 (1.9) | 8.2 (2.1) | 6.6 (3.2) | 7.8 (2.3) | 7.6 (3.1) | 9.3 (6.1) |
| **CRP (mg/ml)** | 4.5 (7.9) | 3.5 (3.7) | 3.4 (5.1) | 5.7 (11.2) | 1.9 (2.0) | 3.1 (3.4) |
| **TGF-β1 (pg/ml)** | 3.0 (1.3) | 3.0 (1.4) | 2.9 (0.9) | 3.2 (1.6) | 3.1 (0.8) | 2.7 (0.9) |

*Note: CLBP- chronic low back pain; CRP- C-reactive protein;* *DASS 21- The Depression Anxiety Stress Scale- 21; IL- interleukin; PCS- Pain Catastrophising Scale; PSEQ- Pain Self-Efficacy Questionnaire; Roland Morris Disability Questionnaire- RMDQ; TNF-α- tumour necrosis factor- α; TGF-β1- transforming growth factor-β1.*
