# Supplementary material for: Systemic pro- and anti-inflammatory profiles in acute non-specific low back pain: An exploratory longitudinal study of the relationship to six-month outcome
Source: PLoS One. 2023 Jun 29;18(6):e0287192. doi: 10.1371/journal.pone.0287192 (PMC10309993; doi:10.1371/journal.pone.0287192)
Supplement: S2 Table — (DOCX) [file pone.0287192.s002.docx]

**Supplementary TABLE S2. Eigenvalues and the degrees of variance (%) of the principal components based on psychological factors at baseline.**

|  | **Eigenvalue** | **% of variance** |
| --- | --- | --- |
| **PC1** | 4.70 | 67.2 |
| **PC2** | 0.70 | 10.0 |
| **PC3** | 0.55 | 7.9 |
| **PC4** | 0.47 | 6.8 |
| **PC5** | 0.30 | 4.3 |
| **PC6** | 0.17 | 2.4 |
| **PC7** | 0.10 | 1.4 |

*Note: PC- principal component.*
